# Cardiorespiratory fitness in breast cancer survivors

**DOI:** 10.1186/2193-1801-2-68

**Published:** 2013-02-25

**Authors:** David Burnett, Patricia Kluding, Charles Porter, Carol Fabian, Jennifer Klemp

**Affiliations:** Breast Cancer Survivorship Center, Mail stop 5015, University of Kansas Medical Center, 3903 Rainbow Boulevard, Kansas City, KS 66103-9815 USA

**Keywords:** Anaerobic threshold, Weight loss, Cardiovascular disease, Chemotherapy, Cardiorespiratory fitness, Exercise testing

## Abstract

Maximal oxygen uptake (VO_2max_) has been used to assess risk for all-cause mortality and cardiovascular disease (CVD), and low VO_2max_ has recently been associated with increased mortality from breast cancer. The purpose of this study was to determine the proportion of breast cancer survivors with 2 or more risk factors for CVD exhibiting a low VO_2max_ and to determine whether sub-maximal endpoints which could be applied more readily to intervention research would correlate with the maximal treadmill test. We performed a single VO_2max_ test on a treadmill with 30 breast cancer survivors age 30–60 (mean age 50.5 ± 5.6 years) who had 2 or more cardiac risk factors for CVD not related to treatment and who had received systemic therapy and or left chest radiation. Submaximal VO_2_ endpoints were assessed during the VO_2max_ treadmill test and on an Arc trainer. Resting left ventricular ejection fraction (LVEF) was also assessed by echocardiogram (ECHO) or multi-gated acquisition scan (MUGA). A majority (23/30) of women had a VO_2max_ below the 20th percentile based on their predicted normal values. The group mean resting LVEF was 60.5 ± 5.0%. Submaximal VO_2_ measures were strongly correlated with the maximal test including; 1) 85% age predicted maximum heart rate VO_2_ on treadmill, (r = .89; p < 0.001), 2) treadmill VO_2_ at anaerobic threshold (AT), (r = .83; p < 0.001), and 3) Arc VO_2_ at AT, (r = .80; p < 0.001). Breast cancer survivors with 2 or more CVD risk factors but normal LVEF had a low cardiorespiratory fitness level compared to normative values in the healthy population placing them at increased risk for breast cancer and cardiovascular mortality. Submaximal VO_2_ exercise testing endpoints showed a strong correlation with the VO_2max_ test in breast cancer survivors and is a good candidate for testing interventions to improve cardiorespiratory fitness.

## Introduction

More than 290,000 women were estimated to be diagnosed with breast cancer in 2012, and the number of survivors has risen beyond 2.5 million (ACS 2012). There is an excess of cardiovascular deaths in breast cancer survivors (Eloranta et al. 2012). Likely due to physical inactivity and weight gain as well as side effects of treatment (Ewer et al. 2011; Jones et al. 2007a). Low cardiorespiratory fitness usually measured by oxygen uptake at maximal or peak exercise (VO2 max or peak) is inversely correlated with cardiovascular and all cause mortality, and breast cancer deaths (Blair et al. 1996; Blair et al. 1989; Peel et al. 2009). Women with low cardiorespiratory fitness (below VO_2max_ of 8 METS (28 mL^.^kg^-1.^min^-1^ VO_2_ or below) had a nearly 3 fold increase in breast cancer deaths compared to those who reached a level above 8 METS (Peel et al. 2009). A recent study indicated breast cancer survivors had a 22% lower VO_2max_ compared to their age matched healthy, sedentary non-cancer peers (Jones et al. 2012).

While a maximal exercise test is the gold standard for assessing cardiorespiratory fitness, a maximal cardiorespiratory exercise test requires participants to push themselves to volitional exhaustion and peak heart rate (HR) (ACSM 2009). In contrast, submaximal testing allows for a measure of sustainable cardiorespiratory exercise capacity (Wasserman et al. 1999), without maximizing heart rate. In addition, submaximal exercise testing is less expensive than maximal testing in that it does not require physician presence during the test.

Submaximal exercise testing has been used to provide a measure of cardiorespiratory fitness, with outcomes based on anaerobic threshold in patients with a history of congestive heart failure (Kemps et al. 2008), stroke (Eng et al. 2004), cancer (Carlson et al. 2006), and athletic populations (Bernardi et al. 2010). Furthermore, submaximal testing is more feasible for use in de-conditioned women who may discontinue a maximal test early because of fatigue - a complaint in 50-80% of breast cancer survivors who have undergone systemic therapy (Andrykowski et al. 1998; Gitt et al. 2002).

A recent meta-analysis indicates a need for a standardized and objective measure of fitness which can be readily utilized in the breast cancer survivorship population (McNeely et al. 2006). Accordingly, we investigated the association between the gold standard measure of cardiorespiratory fitness (VO_2max_) with different submaximal testing endpoints in breast cancer survivors. We hypothesized the VO_2_ at submaximal endpoints would be highly correlated with VO_2max_ in breast cancer survivors and they would have a lower VO_2max_ than age and gender matched normative values. Important clinical implications can arise from evaluating cardiorespiratory fitness in breast cancer survivors and targeting those in the greatest need of preventive measures.

## Materials and methods

### Study participants

This was a cross-sectional study using a within-subject design to investigate the cardiorespiratory fitness and associations between submaximal and maximal cardiorespiratory tests in breast cancer survivors. A total of 30 breast cancer survivors were enrolled in the study. Recruitment was performed by the Breast Cancer Survivorship Navigator/Coordinator from long-term survivorship clinics at the University of Kansas Breast Cancer Survivorship Center (BCSC). Inclusion criteria were as follows: previously diagnosed with stage I-IIIa breast cancer; ages 30–60; no evidence of metastatic disease; at least 3-months from completing initial chemotherapy and/or radiation therapy; and within 10 years of initial diagnosis. Subjects had to also have 2 of the following cardiac risk factors: body mass index (BMI) ≥ 25 kg^.^m^-2^, hypertension (systolic blood pressure ≥ 140 mm Hg and/or diastolic blood pressure ≥ 90 mm Hg),(ACSM 2009) elevated low density lipoprotein (LDL) (fasting serum level ≥ 130 mg^.^dL^-1^) (NCEP 2002), positive family history of myocardial infarction, coronary revascularization, or sudden death in mother, father, or first-degree relative, sedentary lifestyle (not currently participating in habitual exercise of moderate to vigorous intensity for two or fewer sessions per week), smoker (currently smoking or quit smoking within the last six months); and 1 treatment-related risk factor: chemotherapy +/− left breast radiation; and available results of a resting ECHO or MUGA prior to receiving initial chemotherapy. Objectives were to analyze VO_2max_ and submaximal endpoints in breast cancer survivors who had been treated with adjuvant therapy. The submaximal tests utilized a treadmill and an Arc Trainer (Cybex Arc Trainer 750AT). An arc Trainer was used instead of a treadmill in cases where minimization of vertical stress was needed such as women with knee and back problems. Arc Trainers use a low-impact mechanical design with production of lower vertical forces and joint loading compared to walking on a treadmill (Lu et al. 2007; Turner et al. 2010).

The study was approved by the University of Kansas Medical Center Human Subjects Committee (HSC #12251) and written informed consent was obtained from all participants at an orientation meeting prior to conducting any study testing or activities.

### Outcomes

All study procedures were conducted during three testing sessions within a two week time frame at the Clinical Translational Science Unit (CTSU) on the University of Kansas Medical Center campus and the cardiovascular lab located at the University of Kansas Hospital. Participants were instructed to refrain from exercise 48 hours prior to maximal and submaximal cardiorespiratory testing. In addition, all participants were asked not to consume any food, caffeine, alcohol, and tobacco products three hours prior to exercise tests. The exercise testing sessions were between 2 and 14 days apart and at the same time of the day. All cardiorespiratory tests followed the American College of Sports Medicine (ACSM) guidelines for procedures during graded exercise testing (ACSM 2009). In addition, the exercise testing sessions were at least 48 hours, but no more than 14 days, from the resting ECHO/MUGA. Data on demographic information and health history were obtained (Table 1).

**Table 1:** **Non-treatment related characteristics**

Variable	Breast cancer survivors (***n*** = 30)
Age (y)	50.5 ± 5.6
Time since diagnosis (months)	58 ± 27
BMI (kg^.^m^-2^)	29.2 ± 5.3
Body fat (%)	44.5 ± 7.7
Overweight (BMI > 25 kg^.^m^-2^)	25/30 (83%)
Hypertension	8/30 (27%)
↑ LDL	20/30 (66%)
Family history heart disease	12/30 (40%)
Sedentary lifestyle	10/30 (33%)
Smoker	1/30 (3%)

Note: *BMI*, body mass index; *LDL*, low density lipoprotein.

#### Maximal treadmill test

Cardiorespiratory fitness was assessed using a modified Balke protocol (Balke 1959; Blair et al. 1989). Tests were conducted by qualified personnel including an exercise physiologist, nurse, and CTSU medical monitor. An integrated metabolic measurement system (Parvo Medics TrueOne 2400) was used for measurement of maximal oxygen consumption interfaced with an ECG system (Schiller AT-10 Cardiograph). The metabolic cart was set to produce a 15-second average of the data collected during gas analyses for all tests. Equipment was calibrated per manufacturers recommendation prior to testing. Participants were fitted and acclimated to a mouthpiece and headgear (Hans Rudolph; Shawnee, KS) before stepping on to the treadmill. Participants were encouraged to give a maximal effort during the test. The protocol involved a slow progression towards a static speed of 3.3 MPH at the start of minute three, with an initial minute at 2.0 MPH and a second minute at 2.7 MPH. At minute three the speed increased to 3.3 MPH and a grade of 1%. The grade increased 1% each minute thereafter. Participants continued walking on the treadmill at increasing incline until exhaustion, unless indications for terminating the maximal test were observed (ACSM 2009). Participants cooled-down at 1.5-2.0 MPH and a level grade for a period of two to five minutes. Expired air was analyzed for O_2_ and CO_2_. Heart rate, blood pressure, and rating of perceived exertion score were determined at the end of each two minute stage. Confirmation of a maximal effort was determined by meeting three out of four of the following criteria; 1) plateau in heart rate and oxygen uptake with increased workload, 2) respiratory exchange ratio > 1.1, 3) rating of perceived exertion > 17, and 4) heart rate > 90% of age predicted maximal heart rate.

#### Submaximal treadmill test

Cardiorespiratory fitness submaximal endpoints on the treadmill were determined by the VO_2_ at anaerobic threshold and 85% age predicted maximum heart rate. Initial criteria for anaerobic threshold was established by using the respiratory exchange ratio ≥ 1.0. Participants were allowed to continue for three consecutive 15 second averages at a respiratory exchange ratio ≥ 1.0 to ensure anaerobic threshold was reached. Post-test confirmation of the anaerobic threshold was made upon visual assessment of 1) V-slope method, and 2) ventilatory equivalent technique to rule out hyperventilation. Submaximal VO_2_ at 85% age predicted maximum heart rate was assessed with the calculation [(220-age) × 0.85] and determination of the corresponding VO_2_ at that heart rate during maximal testing on the treadmill.

#### Submaximal arc (Cybex 750AT) test

After a brief familiarization trial followed by a short rest period, participants began the Arc submaximal test. Participants’ weight was input into the console before starting the test. The manual mode was used for testing, with initial workload set at a resistance level of 15. Participants were asked to maintain between 80–100 strides per minute and a level grade was used throughout the test. Workload was adjusted to allow a steady increase in heart rate until subjects reached the anaerobic threshold endpoint. Increased workload was performed manually by a gradual increase in wattage while adjusting resistance in two minute stages. Resistance at each two minute stage varied between participants to allow a steady increase in heart rate, and was adjusted by a 5–10 numerical increase on the Arc trainer console resistance key pad. The cardiorespiratory fitness submaximal endpoint on the Arc trainer was determined by the VO_2_ at anaerobic threshold. Criteria for anaerobic threshold was determined by the same method as the submaximal treadmill endpoint.

#### Cardiorespiratory fitness (VO_2max_)

Cardiorespiratory fitness was measured by the participants’ VO_2max_ during the maximal treadmill test. Group mean VO_2max_ was compared to the normative VO_2max_ value for healthy age-matched women (ACSM 2009). We performed our maximal treadmill test with a similar Balke protocol that was used for determining normative VO_2max_ values.

#### Left ventricular ejection fraction

Resting LVEF was performed on all breast cancer survivors by either an ECHO or MUGA, depending on which cardiac function test the participant had prior to starting breast cancer treatment. As previously mentioned, all breast cancer survivor participants had an ECHO or MUGA test within 2 weeks of their cardiorespiratory test. Cardiac function (LVEF) was assessed by a cardiologist not associated with the study.

#### Anthropometrics and body composition

Body weight was assessed to the nearest 0.1 kg, using an electronic scale (Health o meter, Boca Raton, Fl). A stadiometer was used to measure height without shoes to the nearest 0.1 cm (SECA). Body composition was measured by dual-energy x-ray absorptiometry in the total body scanning mode with a Lunar Prodigy DXA machine (Lunar Corp., Madison, WI). Body mass index was calculated as the weight in kilograms divided by the height in meters squared (kg^.^m^-2^).

#### Other study measures

Blood pressure and resting heart rate was measured at rest prior to exercise testing. Family history of heart disease, sedentary lifestyle, and smoking habits was assessed from the chart review, clinical interview, and self-report during initial screening for the study.

### Statistical analysis

Pre-formatted data collection and case report forms were used for both clinical and laboratory data. Study data was entered into an Excel database by a trained data clerk not related to the study. Values and percentages were used to describe VO_2max_ in breast cancer survivors compared to age and gender matched normative values. Distribution normality and was verified using the Shapiro-Wilk test and variance homogeniety was confirmed from a Levene’s test. After assumptions were met, an association between all submaximal VO_2_ endpoints and VO_2max_ was examined using Pearson correlation coefficients. Correlations were categorized as 0.26 to 0.49 is a low correlation, 0.50 to 0.69 is moderate, 0.70 to 0.89 is high, and 0.90 to 1.00 is very high as described by Munro et al. (Munro 1993). All analyses were two-tailed with alpha = 0.05, and performed with SPSS statistical software (version 15.0; SPSS Inc, Chicago, IL).

## Results

### Cohort characteristics

Cohort characteristics and non-treatment CVD risk factors are listed in Table 1. Mean age was 50.5 ± 5.6 years, 29/30 were caucasion, 1/30 was Latino, and the group had an average elapsed time since diagnosis of 58 months. The most frequent non-treatment related risk factors were BMI > 25 kg/m^2^ (83%), elevated LDL (66%), family history of heart disease (40%), and sedentary lifestyle (33%). Additionally, the group mean body fat % was 44.5 ± 7.7%. Treatment related risk factors are listed in Table 2. 28/30 (93%) of the breast cancer survivors were postmenopausal at time of study, while 20/30 (66%) of the women were treated with an aromatase inhibitor. A large majority (80%) of the women received either doxorubicin or epirubicin, 27% were treated with trastuzamab, and 40% had left chest radiation. Also, the group mean LVEF at time of study was 60.5 ± 5%, which was significantly decreased (p = 0.02) from 63.2 ± 5.7% at time of diagnosis and prior to treatment.

**Table 2:** **Treatment related characteristics**

Variable	Breast cancer survivors (***n*** = 30)
Current Aromatase Inhibitor	14/30 (47%)
Current Tamoxifen	1/30 (3%)
Past Aromatase Inhibitors	4/30 (13%)
Past Tamoxifen	1/30 (3%)
Post-menopausal	28/30 (93%)
Adriamycin/Epirubicin	24/30 (80%)
Herceptin	8/30 (27%)
Left chest radiation	12/30 (40%)

### Cardiorespiratory fitness levels

**Figure 1:**
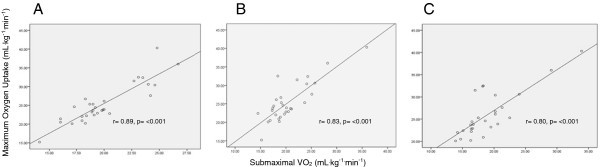
**The association between (A) treadmill 85% age predicted maximum heart rate endpoint, (B) treadmill AT endpoint, and (C) Arc trainer AT endpoint submaximal tests with maximal oxygen uptake.**

All cardiorespiratory fitness measures are listed in Table 3. 23/30 (77%) of the breast cancer survivors with 2 or more CVD risk factors tested below the 20th percentile consistent with low cardiorespiratory fitness based on their age group and gender for VO_2max_ as established by the American College of Sports Medicine (ACSM 2009), despite a mean time since diagnosis of over 4 years and a normal group mean LVEF at time of study. All submaximal testing of VO_2_ endpoints showed a high correlation to the actual measured VO_2max_ when comparing within subject results (Figure 1), and the VO_2_ at 85% age predicted maximum heart rate on the treadmill showed the highest correlation with VO_2max_. Our analysis of cardiorespiratory fitness revealed that the breast cancer survivor group’s mean VO_2max_ (25.4 ± 5.3 mL^.^kg^-1.^min^-1^) was similar to the 20th percentile threshold value (25.1 mL^.^kg^-1.^min^-1^) for age and gender group matched normative values.

**Table 3:** **Cardiorespiratory outcomes**

Variable	Breast cancer survivors (***n*** = 30)
Maximal VO_2_ (mL^.^kg^-1.^min^-1^)	25.4 ± 5.3
Maximal HR (bpm)	169 ± 12
VO_2_ at AT on treadmill (mL^.^kg^-1.^min^-1^)	20.5 ± 4.3
VO_2_ at AT on Arc (mL^.^kg^-1.^min^-1^)	19.0 ± 4.4
VO_2_ at 85% APMHR (mL^.^kg^-1.^min^-1^)	19.9 ± 3.0
HR at AT on treadmill (bpm)	148 ± 13
HR at AT on Arc (bpm)	144 ± 13
HR at 85% APMHR (bpm)	144 ± 5
Predicted Maximal HR (bpm)	170 ± 6
Predicted VO_2_- 85% APMHR (mL^.^kg^-1.^min^-1^)	24.5 ± 7.2
Predicted VO_2max_ (mL^.^kg^-1.^min^-1^)	34.5 ± 8.8
Resting HR (bpm)	78 ± 10
Maximal O_2_ pulse (mL/beat)	11.5 ± 1.5
Pre-Treatment LVEF	63.2 ± 5.7%
Study LVEF	60.5 ± 5.0%

Note: *HR*, heart rate; *AT*, anaerobic threshold; *APMHR*, age predicted maximal heart rate; *LVEF*, left ventricular ejection fractions.

### Adverse events from testing

All 30 maximal and 30 submaximal cardiorespiratory tests were completed without any adverse events. During maximal testing twenty-eight of thirty participants met our established criteria for a maximal effort as explained previously. Two of the participants discontinued the test secondary to a claustrophobic sensation related to the mouthpiece, fatigue, and/or shortness of breath. Symptoms were resolved by discontinuing the test and having the participants rest while monitoring all post-test vitals. Even though two participants discontinued the maximal test prior to reaching maximal criteria, both participants achieved > 85% APMRH on the treadmill and have been included in the analyses.

## Discussion

In our breast cancer survivors cohort, all participants had ≥ 2 risk factors for CVD not related to treatment and 77% of this group had low cardiorespiratory fitness measured by VO_2max_ despite a normal LVEF at time of study. These results suggest that they are at a higher risk of breast cancer and cardiovasular mortality (Blair et al. 1996; Blair et al. 1995; Peel et al. 2009). Our results are similar to three earlier reports in breast cancer survivors, (one of which women had controlled hypertension), suggesting the majority of women with non-treatment related CVD risk factors have a lower VO_2max_ compared to healthy women even with a concomitant normal LVEF (Jones et al. 2012; Jones et al. 2007b; Tolentino et al. 2010). Our study differs from the earlier studies since we used a similar VO_2max_ protocol (Balke protocol) to the standardized treadmill test used for determining normative values (ACSM 2009) and we reported pre- and post-treatment LVEF.

Collectively, the double impact from the effects of lifestyle changes and breast cancer adjuvant treatment may increase the risk for *late-onset* CVD. Despite this knowledge, there is not a current stratification tool to accurately assess increased risk of CVD morbidity and mortality in breast cancer survivors. For example, sub-clinical cardiac dysfunction may go unnoticed until more overt symptoms occur and still remain undetected by a resting echocardiogram (ECHO) (Cardinale et al. 2004; Civelli et al. 2006). However, exercise tests may be more sensitive than resting tests in identifying cardiac dysfunction in long-term survivors (Gottdiener et al. 1981; Klewer et al. 1992; Weesner et al. 1991). Therefore, exercise testing may serve as an important clinical tool for identifying breast cancer survivors who are asymptomatic, but at increased risk for the development of CVD. Furthermore, cardiorespiratory exercise testing can provide an objective evaluation of cardiorespiratory fitness, reducing the variability found in self-reported activity measures by 70-80% (Blair & Church 2004).

Sub-maximal exercise testing with VO_2_ measured at the anaerobic threshold has shown good correlation with maximal exercise testing in individuals where a maximum test would be difficult because of disability or de-conditioning including those with congestive heart failure, stroke, or undergoing bone marrow transplant (Carlson et al. 2006; Eng et al. 2004; Kemps et al. 2008). To our knowledge, this is the first study to investigate the association between maximal and submaximal cardiorespiratory fitness testing in breast cancer survivors. We found that submaximal VO_2_ endpoints were highly correlated with VO_2max_, indicating that submaximal testing can be a good measure of cardiorespiratory fitness in breast cancer survivors. Our study produced similar results when comparing submaximal VO_2_ at anaerobic threshold and 85% age predicted maximum heart rate to VO_2max_, suggesting that submaximal testing can be used as a surrogate for VO_2max_ testing in breast cancer survivors.

The submaximal VO_2_ at 85% age predicted maximum heart rate was used because this predetermined endpoint can be performed without expensive gas analysis equipment and can be more feasible than measuring anaerobic threshold when a large number of patients or subjects need to be tested. Submaximal VO_2_ endpoint at 85% age predicted maximum heart rate had a similar group mean heart rate and VO_2_ compared to the anaerobic threshold endpoint. This is important to note since anaerobic threshold is a helpful indicator for determining fitness level and for measuring the effect of exercise training (Casaburi 1994; Casaburi et al. 1991). Overall, the submaximal VO_2_ endpoint at 85% age predicted maximum heart rate showed the highest correlation to actual measured VO_2max_, as seen in Figure 1. Our findings suggest using the speed and grade at the 85% age predicted maximum heart rate endpoint during a submaximal test for predicting VO_2_ and objectively measuring cardiorespiratory fitness for breast cancer survivors, especially when repeated measures are required for assessing improvement after an exercise intervention. Our results support the use of a submaximal cardiorespiratory test as an objective measure of fitness that can be used for breast cancer survivors. Furthermore, this study suggests that a submaximal cardiorespiratory test using a modified Balke protocol with an endpoint set at 85% age predicted maximum heart rate can be performed when the equipment and personnel needed to conduct directly measured oxygen uptake via indirect calorimetry are not available. A validation study to design an accurate predictive model for extrapolating VO_2max_ from a submaximal testing end point at the 85% age predicted maximum heart rate endpoint.

We are unaware of previous studies that have used an Arc trainer with decreased load bearing force as an exercise testing modality to examine cardiorespiratory fitness in breast cancer survivors, many of whom have age-related or aromatase inhibitor induced arthalgia and may prefer alternate forms of testing. Turner et al. showed that VO_2max_ and time to attain VO_2max_ were similar when comparing results between testing modalities including an Arc trainer and a treadmill in healthy adults (Turner et al. 2010). During our study three participants were unable to complete testing on the Arc trainer due to their inability to coordinate the movement between the lower and upper limbs simultaneously. Also, the Arc trainer produced significantly lower VO_2_ and heart rate at anaerobic threshold versus the treadmill submaximal test (Table 3). Overall, we suggest that the benefit of using an Arc trainer does not outweigh difficulty with performing the required coordinated movements and lack of standard testing protocol. Also, It is important to note that there were no breast cancer survivors in our study that could not complete testing on the treadmill due to joint or muscle pain.

This is the first study to investigate the association between the gold standard for cardiorespiratory fitness (VO_2max_) to submaximal VO_2_ tests in breast cancer survivors who had been treated with adjuvant therapy. The findings from this study indicate that breast cancer survivors with ≥ 2 CVD risk factors had low cardiorespiratory fitness and submaximal testing on the treadmill is a feasible, objective measure of fitness that can be used in breast cancer survivors. Limitations of this study include the small sample size (n = 30), lack of racial diversity, and lack of non-cancer controls.

Future research should focus on using a high-risk breast cancer control group with ≥ 2 CVD risk factors to compare maximal and sub-maximal VO_2_ measures. In addition, novel approaches should be made to improve cardiorespiratory fitness during sustainable exercise interventions for breast cancer survivors.

### Ethical standards

All work described within this manuscript complies with United States and institutional regulations for the protection of human subjects.

## Authors’ information

Co-authors: Patricia Kluding, Charles Porter, Carol Fabian, Jennifer Klemp.
